# Impact of COVID-19 on vocal cord mobility: a case series study

**DOI:** 10.1186/s43163-021-00157-y

**Published:** 2021-09-17

**Authors:** Sameh M. Zamzam, Rania Gamal Hanafy

**Affiliations:** 1 ENT Department, Faculty of Medicine, Cairo University, Cairo, Egypt; 2 ENT Department, Kasr Alainy Hospital, Garden City, Cairo, Egypt; 3 ENT Department, Railway Hospital, Ramsis, Cairo, Egypt

**Keywords:** COVID-19, Vocal cord, Paralysis, China, WHO, Otolaryngology

## Abstract

**Background:**

The World Health Organization (WHO) has declared the pandemic of COVID-19 infection in March 2020, most of cases presented with mild symptoms, and a significant number of cases showed variable neurological pictures. Vocal cord paralysis with no clear cause is termed as idiopathic vocal cord paralysis and supposed to be caused by viral infection. This is a case series study; data were collected prospectively from patients presented to the ENT clinic of Kasr Alainy (Cairo university) and Railway hospitals. Patients presented with defective vocal cord movement with concurrently or recently passed COVID-19 infection were reported from March 2020 to April 2021.

**Results:**

Authors have reported 6 cases of vocal cord paralysis mainly unilateral due to COVID-19 infection as an only clear cause within 14 months. Age ranges from 39 to 69 years, 2 males and 4 females. Patients presented with different clinical scenarios. Follow-up of the cases showed spontaneous recovery in 5 cases and one case underwent cord medialization.

**Conclusion:**

Viral infection could be an underlying cause of idiopathic laryngeal cord paralysis; in the new era of the COVID-19 pandemic, physicians all over the world noticed variable neurological pictures; in this study, we presented 6 cases of vocal cord paralysis mainly unilateral supposed to be due to COVID-19 infection; all cases showed spontaneous recovery apart from one case that needed medialization of the cord.

## Background

The World Health Organization (WHO) has declared the pandemic of COVID-19 infection in March 2020. The disease has been arisen in Wuhan, China, and caused by respiratory droplets that carry SARS-CoV-2 [1]. The new disease is challenging due to different clinical scenarios, and most of the cases presented mild symptoms like fever, myalgia, and dry cough [2]. Serious progress of the disease may result in complications such as respiratory failure, disseminated intravascular coagulopathies, and other system failures [3], and a significant number of cases showed different neurological pictures [4] like hyposmia, ageusia, Bell’s palsy, Guillain-Barré syndrome, and encephalopathy [5–8].

Paralysis of the vocal cords is a serious problem; most of the causes are well known apart from some cases that presented with cord paralysis with no clear cause [9, 10]. Authors termed this condition as idiopathic vocal cord paralysis and supposed to be occurred due to viral infection [11, 12].

In this study, authors have spotted the light on a number of cases of laryngeal cord paralysis mainly unilateral as a result of COVID-19 infection.

## Methods

A case series study, data were collected prospectively from patients presented to the ENT clinic of Kasr Alainy (Cairo university) and Railway hospitals. Patients who presented with defective vocal cord movement with concurrently or recently passed COVID-19 infection were reported from March 2020 to April 2021.

## Results

Authors have reported 6 cases of vocal cord paralysis mainly unilateral due to COVID-19 infection as an only clear cause (Table 1).

**Table 1:** Data of study cases

	Case 1	Case 2	Case 3	Case 4	Case 5	Case 6
**Gender**	F	F	M	F	F	M
**Age in years**	48	39	41	51	44	69
**Laryngeal symptoms**	Chocking Dysphonia	Dysphonia	Dysphonia	Dysphonia	Dysphonia	Dysphonia Chocking
**Other symptoms**	Fever Myalgia Bone aches	Fever Bony aches Anosmia Hypogeusia Dry cough	Fever Myalgia Bone aches	No	Fever Anosmia Dyspepsia Nausea Cough	Fever Myalgia Bone aches Cough
**Cord affected**	Right	Right	Left	Both	Left	Right
**Time interval between laryngeal and other COVID symptoms**	Same day of onset	Same day of onset	3 weeks	Not applicable	Same day of onset	Same day of onset
**Swab and PCR**	N/A	Positive	N/A	Positive	N/A	N/A
**CT scan on chest**	Ground glass opacities	Ground glass opacities	Ground glass opacities	Ground glass opacities	Ground glass opacities	Ground glass opacities
**Laboratory data**	Lymphopenia, elevated CRP, D-dimer, serum ferritin, and LDH	N/A	Lymphopenia, elevated CRP, D-dimer, serum ferritin	N/A	Lymphopenia, elevated CRP, D-dimer, serum ferritin	Lymphopenia, elevated CRP, D-dimer, serum ferritin
**Co-morbidities**	No	No	No	Obesity	No	Hypertension
**Endotracheal intubation**	No	No	No	No	No	No
**Treatment of COVID-19**	Home isolation + medical protocol	Isolation hospital	Home isolation + medical protocol	Isolation hospital	Isolation hospital	Isolation hospital
**Received vaccine**	No	No	No	No	No	No
**Follow-up**	Needed cord medialization	Spontaneous recovery	Spontaneous recovery	Spontaneous recovery	Spontaneous recovery	Spontaneous recovery
**Presence of another clear cause of vocal cord paralysis**	No	No	No	No	No	No

*N/A* not available

### Case 1

**Fig. 1:**
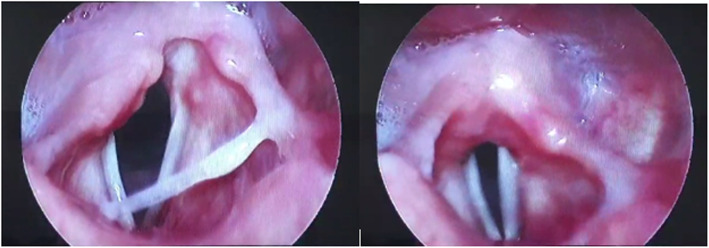
Right vocal cord paralysis by fibro-optic laryngoscopy (case 1)

A lady, 48 years old, presented with hoarseness of voice as a chief complaint and chocking of 3-week duration; by using a flexible fiber optic laryngoscope, we detected right vocal cord paralysis and no laryngeal or neck masses. She gave a history of recent COVID-19 infection manifested by fever, myalgia, bone aches, and fever and no anosmia or ageusia. The onset of these symptoms was at the same day of onset of the laryngeal symptoms. She also gave a negative history of hospitalization or endotracheal intubation. Included diagnostic tools revealed bilateral ground glass opacities in the CT scan on the chest and lymphopenia, elevated C reactive protein (CRP), D-dimer, serum ferritin, and lactate dehydrogenase (LDH). A new CT scan carried out on the skull, neck, and upper half chest was clearly free from any masses (Fig. 1).

### Case 2

**Fig. 2:**
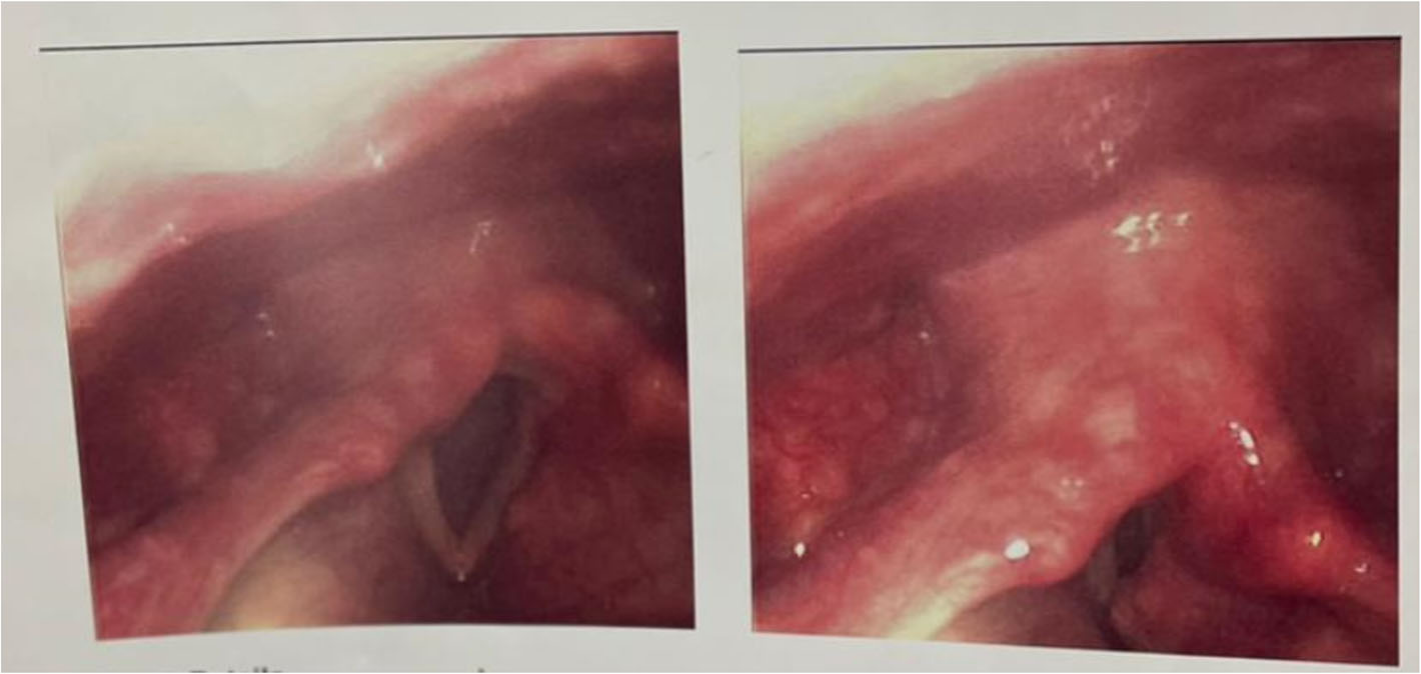
Right vocal cord paralysis (case 2)

Another lady, 39 years old, coming with a history of a 5-day duration of fever, bony aches, anosmia, hypogeusia, dry cough, and change of voice. Nasopharyngeal swab and PCR for COVID-19 and CT scan on the chest were carried out and the results were confirmatory. She was isolated and hospitalized for treatment. Flexible fibro-optic laryngoscopic examination was performed after 3 weeks when the swab turned negative and it was diagnostic for right cord paralysis. A new CT carried out on the skull, neck, and upper half chest was clearly free from any masses (Fig. 2).

### Case 3

**Fig. 3:**
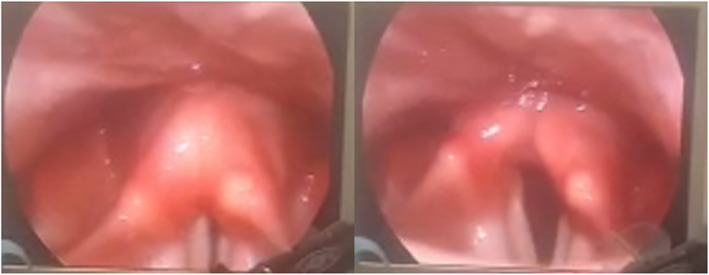
Left vocal cord paralysis (case 3)

A gentleman, 41 years old, past history of COVID-19 infection 5 weeks ago and hoarseness of voice 2 weeks ago. Left vocal cord paralysis was diagnosed by a fibro-optic laryngoscope (Fig. 3).

### Case 4

**Fig. 4:**
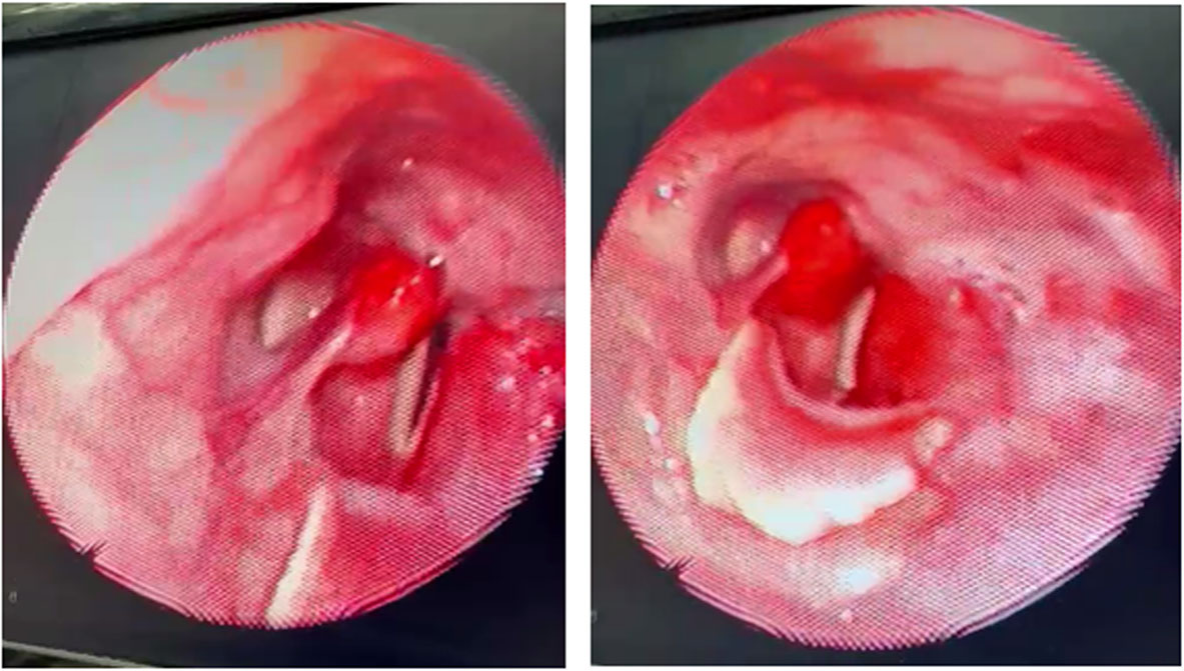
Left vocal cord paralysis and right cord paresis (case 4)

A 51-year-old lady presented with just a mild degree of noisy difficult breathing of 5 days with no other symptoms; examination showed left vocal cord paralysis and right cord paresis. No history of neck surgery was found; a CT scan on the chest, neck, and skull base was carried out as a routine in cord paralysis cases but it showed COVID-19 opacities by accident. Then, she referred for nasopharyngeal swab and PCR that gave a positive result for COVID-19 (Fig. 4).

### Case 5

**Fig. 5:**
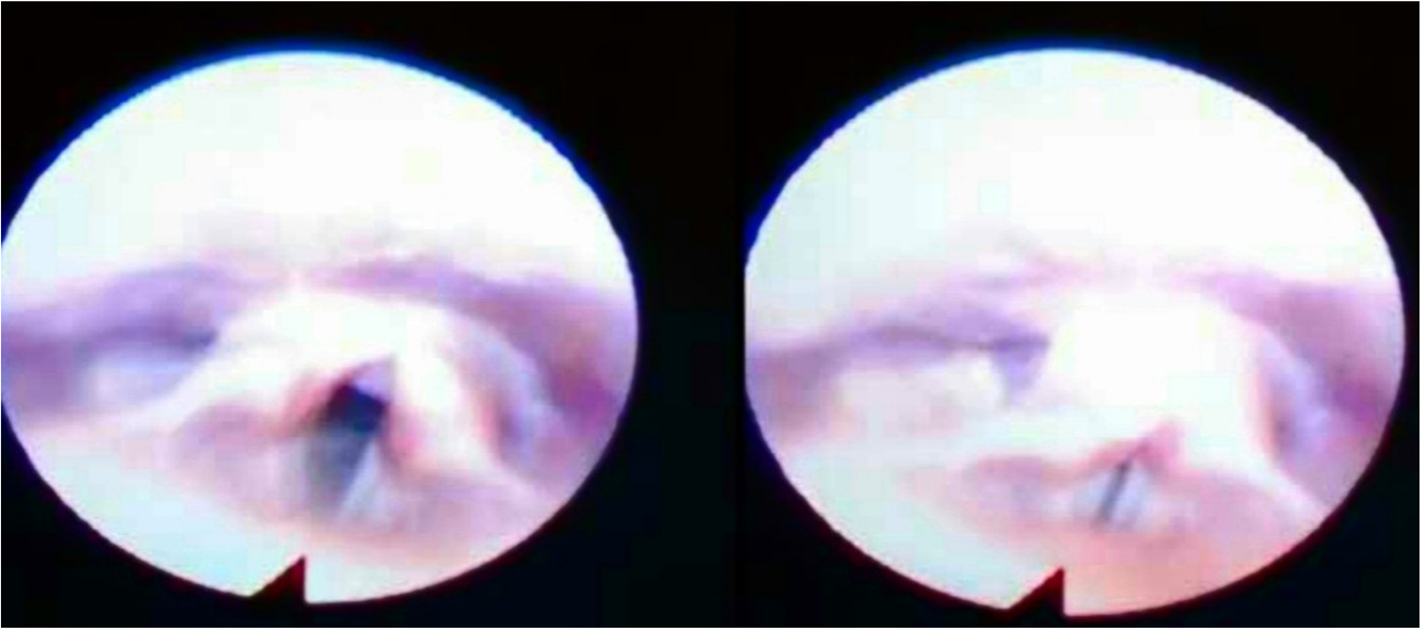
Left vocal cord paralysis (case 5)

A lady, 44-year-old patient, coming to the clinic with fever, anosmia, mild abdominal dyspepsia, nausea, cough, and change of voice 3 days ago. Diagnosis confirmed as COVID-19; after treatment in an isolation hospital and being negative by PCR, the larynx was examined and left vocal cord paralysis was diagnosed (Fig. 5).

### Case 6

**Fig. 6:**
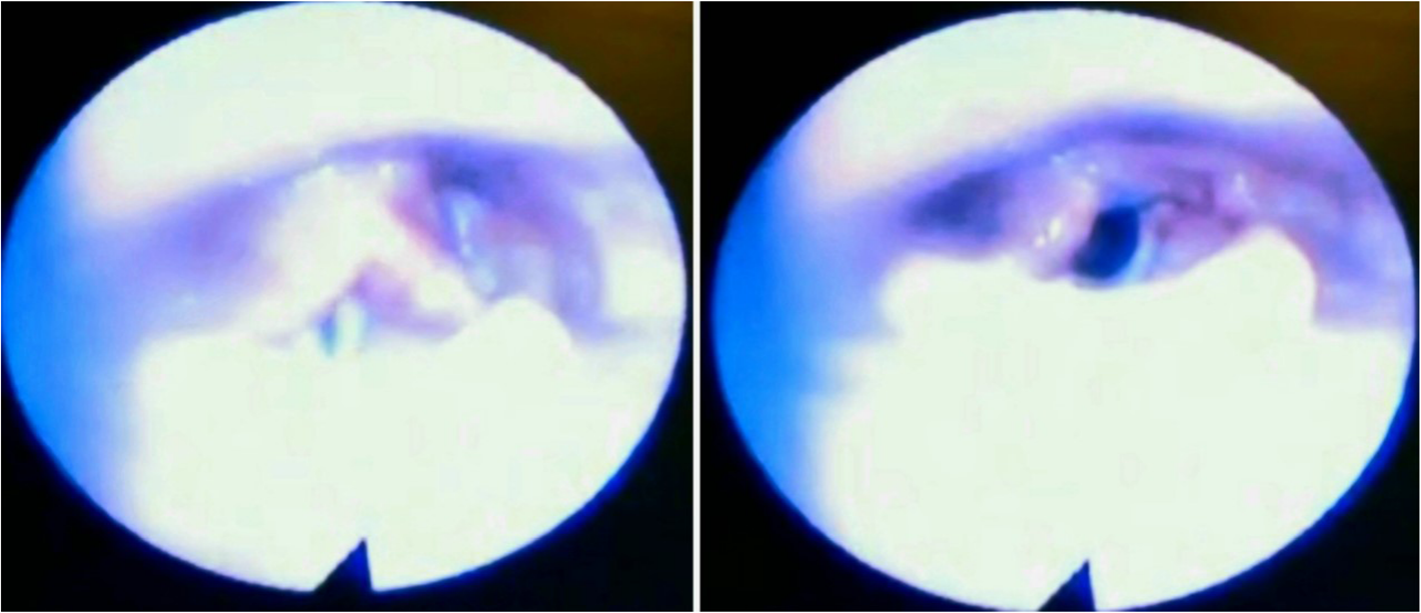
Right vocal cord paralysis (case 6)

A 69-year-old man already referred from an isolation hospital after finishing his treatment from COVID-19. The cause of referral was the persistent cough and change of voice despite a clear recent CT scan on the chest and negative post-treatment PCR. Laryngeal examination showed right vocal cord paralysis (Fig. 6).

All cases started speech therapy and were monitored by regular visits every 2 weeks. Five cases showed variable degrees of spontaneous recovery within 2–5 months; vocal cord medialization by hyaluronic acid was carried out in one case who showed no spontaneous improvement.

## Discussion

This study suggests an association between the COVID-19 infection and the laryngeal vocal cord paralysis as there is no other clear cause of vocal cord paralysis in this study’s cases; none of the study cases was diabetic; also, all cases showed nearly the same onset of COVID-19 symptoms and laryngeal paralysis. Bhatt et al. have suggested the same theory of association between cord paralysis and viral upper respiratory tract infection [13].

Some authors have noticed that the incidence of idiopathic vocal cord paralysis is higher in winter due to the higher spread of droplet infections of viral origin [14–16].

Previous publications have diagnosed specific virus infections like herpes simplex, Epstein-Barr, West Nile, and varicella-zoster viruses clinically and laboratory in cases with idiopathic cord paralysis [12, 17–21].

There are many examples of viral-induced neuropathy in the field of otolaryngology other than the larynx. Anosmia and ageusia became very popular with COVID-19 infection [6, 8], also Bell’s palsy although its etiology is unknown but herpes simplex virus is highly accused as a cause in patients of Bell’s palsy, the same with the varicella-zoster virus which is assumed to be responsible for Ramsay Hunt syndrome [22–25].

The incidence of post-COVID-19 vocal fold paralysis is relatively low; however, mild paresis may be discovered accidentally. Furthermore, laryngeal examination has been limited by the pandemic. Thus, the prevalence, severity, and consequence of vocal cord insult during the COVID-19 pandemic remain to be determined [26]. In a study on 20 patients with post-COVID-19 dysphonia, 40% of cases were diagnosed as unilateral vocal cord paralysis [27]. Another study has reported 2 cases of bilateral vocal cord paralysis due to viral infection by COVID-19 [28].

Finally, literature has focused on anosmia and ageusia as a recently common picture of COVID-19 infection and it lacks studies that entail the issue of COVID-19 impact on vocal cord innervation and mobility.

## Conclusions

Viral infection could be an underlying cause of idiopathic laryngeal cord paralysis; in the new era of the COVID-19 pandemic, physicians all over the world noticed variable neurological pictures; in this study, we presented 6 cases of vocal cord paralysis mainly unilateral supposed to be due to COVID-19 infection; all cases showed spontaneous recovery apart from one case that needed medialization of the cord.

## Data Availability

Data are available from the corresponding author on reasonable request.

## Abbreviations

COVID-19: Coronavirus disease 2019
