# Correction to: Impact of COVID-19 on vocal cord mobility: a case series study

**DOI:** 10.1186/s43163-021-00180-z

**Published:** 2021-11-12

**Authors:** Sameh M. Zamzam, Rania Gamal Hanafy

**Affiliations:** 1 ENT Department, Faculty of Medicine, Cairo University, Cairo, Egypt; 2 ENT Department, Kasr Alainy Hospital, Garden City, Cairo, Egypt; 3 ENT Department, Railway Hospital, Ramsis, Cairo, Egypt

**Corrected to: Egypt J Otolaryngol 37, 93 (2021)**

**https://doi.org/10.1186/s43163-021-00157-y**

Following the publication of the original article [1], the authors identified errors in the affiliations of Dr. Sameh M. Zamzam.

The correct affiliation is given below:

1ENT Department, Faculty of Medicine, Cairo University, Cairo, Egypt

The original article [1] has been corrected.
